# Combination Therapy of PPAR*γ* Ligands and Inhibitors of Arachidonic Acid in Lung Cancer

**DOI:** 10.1155/2008/750238

**Published:** 2009-03-04

**Authors:** Jordi Tauler, James L. Mulshine

**Affiliations:** Section of Medical Oncology, Department of Internal Medicine, Rush University Medical Center, Chicago, IL 60612, USA

## Abstract

Lung cancer is the leading cause of cancer death in the United States and five-year survival remains low. Numerous studies have shown that chronic inflammation may lead to progression of carcinogenesis. As a result of inflammatory stimulation, arachidonic acid (AA) metabolism produces proliferation mediators through complex and dynamic interactions of the products of the LOX/COX enzymes. One important mediator in the activation of the AA pathways is the nuclear protein PPAR*γ*. Targeting LOX/COX enzymes and inducing activation of PPAR*γ* have resulted in significant reduction of cell growth in lung cancer cell lines. However, specific COX-inhibitors have been correlated with an increased cardiovascular risk. Clinical applications are still being explored with a novel generation of dual LOX/COX inhibitors. PPAR*γ* activation through synthetic ligands (TZDs) has revealed a great mechanistic complexity since effects are produced through PPAR*γ*-dependent and -independent mechanisms. Furthermore, PPAR*γ* could also be involved in regulation of COX-2. Overexpression of PPAR*γ* has reported to play a role in control of invasion and differentiation. Exploring the function of PPAR*γ*, in this new context, may provide a better mechanistic model of its role in cancer and give an opportunity to design a more efficient therapeutic approach in combination with LOX/COX inhibitors.

## 1. INTRODUCTION

Lung cancer is the leading cause of cancer death in the United States. Despite increasing amount of effort on lung cancer research, five-year survival is still around 15% [1].

Growing evidence suggests that molecular pathways involved in chronic inflammation may contribute the progression of carcinogenesis [2, 3]. Arachidonic Acid (AA) metabolism is intimately involved in the inflammatory response. Its deregulation in epithelial cancers has been regarded as an early step in the transformation process [4, 5]. AA is released from the membrane by phospholipidic enzymes, mainly through cPLA2*α* activity [6]. AA could be metabolized by two major pathways: lipoxygenase (LOX) pathway producing hydroxy derivatives and leukotrienes and the cyclooxygenase (COX) pathway producing various prostaglandins. Overexpression of LOX and COX enzymes has long been associated with tumor progression [6–8], and targets for those pathways have been a primary interest in research for therapeutics agents [9–11]. However, specific COX-2 inhibitors have been associated with cardiovascular toxicity [12–15]. It has also been reported that products of the LOX pathway and inhibitors of this pathway may induce activity of peroxisome-proliferator-activated receptor gamma (PPAR*γ*) [9, 16, 17].

PPAR*γ* is a member of the nuclear-hormone-receptor superfamily characterized as having a role in lipid metabolism and adipose differentiation [18]. Several synthetic ligands activate PPAR*γ* and reduce cell growth by inducing apoptosis in lung cancer [19, 20]. Combination therapy of 5-LOX inhibitor, PPAR*γ* ligand, and PPAR*γ* binding partner has resulted in an additive effect on cell growth decrease and induction of apoptosis [21]. However, synthetic PPAR*γ* ligands such as thiazolidinedione derivatives (TZDs), a class of antidiabetic drugs, are also responsible of PPAR*γ*-independent effects [22, 23]. TZDs compounds (pioglitazone, rosiglitazone, trosiglitazone, ciglitazone) have shown interesting clinical activity in diabetes and metabolic syndrome but also have been associated with rare but significant clinical toxicities [24, 25].

New COX/LOX inhibitors have been recently reported and initially these drugs showing a more favorable GI and cardiovascular tolerability [26]. Exploring the potential of these new agents, together with a more comprehensive mechanistic model of the PPAR*γ* function, may provide a solid foundation for a better design of novel combination therapies for lung cancer.

## 2. INHIBITION OF LOX/COX PATHWAYS

Two isoforms of (COX) enzymes have been identified and targeted for their clinical and pharmacological interest [27, 28]. Characterization of COX-1 and COX-2 enzymes led to a proposed model where COX-1 was constitutively expressed and COX-2 was an inducible enzyme activated in inflammatory response [29, 30]. Overexpression of inducible COX-2 has also been reported in malignant conditions associated to cell growth, protection against apoptosis, and induction of angiogenesis in lung cancer [31–34]. Selective COX-2 inhibitors have reduced cell growth and increased apoptosis in lung cancer cell lines [32, 35, 36]. However, increased cardiovascular risk has been associated with selective inhibition of COX-2 [12–15].

LOX pathway is more complex since at least six different enzymes have been identified in humans, and it has not been as extensively developed for clinical applications [37]. Studies of LOX expression and activities in normal and cancerous lesions have shown that 15-LOX-1 and 15-LOX-2 are usually expressed in normal tissues and benign lesions, whereas 5-LOX and 12-LOX are absent in normal epithelia and constitutively expressed in epithelial cancers such as lung, colon, skin, esophageal, pancreatic, and prostate cancers [38]. Targeting 5-LOX with specific inhibitors or by inhibition of 5-lipoxygenase activating protein (FLAP) has resulted in decreased cell growth and increased apoptosis in lung and breast cancer cell lines [39]. In that report, 5-LOX downstream metabolites were reduced due to a diversion of the metabolic products from 5-LOX to other LOX (12-LOX and 15-LOX) and COX pathways. Substrate of inhibited 5-LOX is metabolized by the other available enzymes of both LOX and COX pathways. This result has been described as endoperoxide shunting [39]. This property of the AA pathways adds another layer of complexity to this mechanism. To address this complexity, Hong et al. [40] analyzed, in epithelial cancer cell lines, the correlation between expression of AA metabolizing enzymes and effect on cell growth of specific enzyme inhibitors. No correlation was observed for inducible enzymes (LOX-12, LOX-15, and COX-2). However, LOX inhibitors have a more potent effect on cell growth in vitro than COX inhibitors on constitutively expressed enzymes, LOX-5, and COX-1 [40]. Of interest, pan-COX inhibitor ketorolac did not inhibited oral cancer growth in vitro, but it was associated with significant reduction of heterotransplant growth in vivo [11]. Cytokine-producing inflammatory cells are present in the in vivo assay. Stimulated macrophages and other inflammatory cells are able to produce a variety of cytokines which could promote growth differentially on clonal populations of epithelial cells. Hong et al. [11] have suggested that IL-6 plays, through STAT3 signaling, an important role in oral cancer regulation in a paracrine and autocrine way. This report suggests a potential role for inflammatory cells stimulating cancer cell growth by COX-driven cytokine production.

Recent animal studies have shown COX-2 constitutive expression in normal tissues, where it plays a role in gastric mucosal protection, renal homeostasis, and endothelial PGI_2_ production [41, 42]. This result, along with the previously described risk of thrombotic complication after selective inhibition of COX-2, motivated the search for an alternative strategy [43]. Since inhibition of one pathway of the AA metabolism might induce the activity of the alternate pathway, a dual inhibition of both LOX and COX pathways has been proposed as a new approach to improve clinical utility [44]. Moreover, inhibition of COX-2 increases production of leukotrienes (LTs), especially in the gastric mucosa. Given the proinflammatory effects of LTs and their deleterious effects on gastric mucosa, dual inhibition of LOX and COX pathways might improve gastric tolerability [45]. On the other hand, free unmetabolized AA may induce a concentration-dependent apoptosis on cancer cells [46]. Therefore, blocking LOX/COX pathways simultaneously may prevent recruitment of alternate pathways within the AA pathway and may lead to an accumulation of AA that could increase apoptosis induction.

The use of combination of LOX and COX specific inhibitors has been described in colon and pancreatic cancer models [47, 48]. Recently, Schroeder et al. [49] have reported that treatment of A549 lung cancer cell line and transformed cell 1198, derived from BEAS-2B, with a triple combination of clinical relevant concentrations of celecoxib (COX inhibitor), MK886, and REV 5901 (both LOX inhibitors) resulted in significant suppression of growth and cell death induction in both cell lines. Interestingly, premalignant cells, derived from BEAS-2B, revealed a greater sensitivity to this LOX/COX inhibitors combination than malignant cells A549. This result raises the possibility that combination of AA metabolism inhibitors might be more effective in precancerous states than in lung cancer therapy.

However, designing a single compound that might target both LOX and COX pathways is a strategy that offers several benefits in terms of cost, risk, and adverse effects [50]. By using different approaches, a number of new compounds able to target LOX-5 and COX-2 have been designed but to date limited data is available concerning their potential as antitumorigenic agents [51]. First generation of compounds showing dual inhibition of LOX-5 and COX-2 such as Benoxaprofen is not longer in use due to their liver toxicity [52]. A new generation of compounds has been developed offering a more balanced inhibition of LOX-5 and COX-2 enzymes by acting as a substrate competitor. Licofelone is one of the most promising candidates and is currently on phase-III clinical trials for treatment of osteoarthritis as anti-inflammatory drug [26]. Licofelone inhibits LOX-5, COX-1, and COX-2, decreases production of PGs and LTs [53, 54], and presents lower GI toxicity compared to nonsteroidal anti-inflammatory drugs (NSAIDs) naproxen and rofecoxib [55, 56]. Interestingly, it has been reported recently that Licofelone inhibits LOX/COX pathways and induces apoptosis in HCA-7 colon cancer cells [57].

## 3. PPAR*γ* ACTIVATION

Active PPAR*γ* forms a heterodimer with retinoid X receptor RXR [22, 58, 59]. Coactivators and corepressors interact with the PPAR*γ*-RXR heterodimer which binds specific regions known as PPRE (PPAR*γ* response elements) within promoter of target genes. Different interactions of coactivators and corepressors with PPAR*γ* are responsible for important changes on the transcription pattern of target genes. Some natural ligands of PPAR*γ* have been identified such as leukotrienes, prostaglandin D_2_, prostaglandin J_2_ (15d-PGJ_2_), and some polyunsaturated fatty acids. In addition, antidiabetic drugs, such as rosiglitazone, ciglitazone, pioglitazone, and trosiglitazone, included in the group of thiazolidinediones (TZDs), are also ligands of PPAR*γ*. Activation of PPAR*γ* in NSCLC by TZDs has induced cell growth arrest and apoptosis [60, 61] and affects expression of genes such as PTEN, fibronectin, and integrin alpha 5 [62–64]. However, PPAR*γ* ligands have shown effects on NSCLC cell lines that remain elusive to understand with our current notions of PPAR*γ* mechanism of action. For instance, rosiglitazone inhibits cell growth by increasing phosphorylation of AMPK*α* and reducing phosphorylation of p70S6K. Treatment with PPAR*γ* antagonist, GW9662, has no effect on AMPK*α* and p70S6K phosphorylation status [65].

PPAR*γ* could also be regulated from upstream elements of the AA pathway; interestingly, it has been reported that cPLA2*α*, responsible of AA release from the membrane, affects PPAR*γ* activity and modulates expression of COX-2 and IL-8 through PPAR*γ* response elements [66, 67].

## 4. LOX/COX PATHWAYS AND PPAR*γ* CROSS-TALK

**Figure 1:**
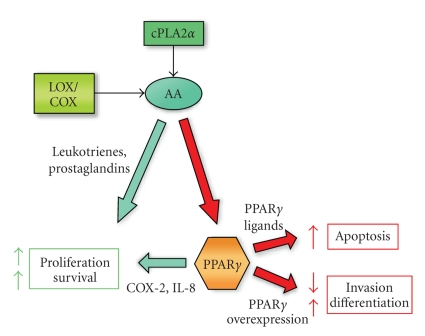
Inflammatory products stimulate cPLA2*α* which increases levels of AA available to be metabolized by the LOX/COX pathways. LOX/COX enzymes' products, leukotrienes and prostaglandins, induce cell proliferation. Natural ligands for PPAR*γ* are also produced by the LOX/COX pathways. Activation of PPAR*γ* balances the effect of leukotrienes and prostaglandins inducing apoptosis through various mechanisms. However, activation of PPAR*γ* may also induce COX-2 and IL-8 expressions. Furthermore, overexpression of PPAR*γ* may decrease invasion and induce differentiation from a mesenchymal to an epithelial-like phenotype.

Deregulation of important elements of the AA pathway has been observed in tumor progression in several reports as shown in Figure 1 [4, 5]. Targeting overexpression of inducible enzymes, 5-LOX and COX-2 enzymes, as a way to control cell proliferation was a first logical step. However, as we have previously discussed, downregulation of one pathway of the AA metabolism may induce, through endoperoxide shunting, the other AA pathways, balancing the initial effect [68–72].

Affecting the LOX/COX pathway has also an effect on the activity of PPAR*γ*. Inhibition of 5-LOX by MK886 results in activation of PPAR*γ* in breast and lung cancer cell lines [9, 21]. Inhibition of COX-2 by celecoxib reduces PGE2 production, downregulates cPLA2*α* expression in lung cancer cell lines, but also induces PPAR*γ* expression and activity [73, 74]. On the other hand, PPAR*γ* ligand ciglitazone may modulate COX-2 expression and PGE2 production through a PPAR*γ*-independent mechanism. Thus, it has been speculated that ciglitazone could suppress transcriptional factors involved in COX-2 mRNA production. It has also been suggested that ciglitazone could downregulate COX-2 through a histone deacetylase mechanism [75]. Furthermore, PPAR*γ* ligands rosiglitazone and pioglitazone decrease PGE2 by upregulating 15-hydroxyprostaglandin dehydrogenase independently of PPAR*γ* and COX-2 [76].

MK886, inhibitor of FLAP (5-lipoxygenase activating protein), results in inhibition of 5-LOX and induction of PPAR*γ* activity [9]. Induction of PPAR*γ* might be a direct effect of MK886 [77] or an indirect effect as a result of changes in the equilibrium of AA available for each different LOX enzymes after inhibition of 5-LOX. Thus, production of 15-HETE might increase which could induce PPAR*γ* expression [16, 17]. An interesting example of the double effect, inhibition of 5-LOX and activation of PPAR*γ*, has been provided by Avis et al. [21] showing that a combination of low-dose MK886, ciglitazone (PPAR*γ* ligand), and retinoic X receptor (RXR*α*; transcriptional partner of PPAR*γ*), interacting in a superadditive manner, causes an inhibition of cell growth in lung cancer cell lines A549 and H1299. Moreover, a novel compound, LY293111, an LTB_4_ receptor antagonist and inhibitor of 5-LOX, is able to induce PPAR*γ* as well [78]. LY293111 has proved to be effective in reducing cell growth in different types of cancer such as pancreatic, colon, and lymphoma [79–83]. However, no results have been published so far in lung cancer.

Induction of PPAR*γ* by 15-LOX metabolites and by COX-2 inhibitors and PPAR*γ* effects on COX-2 activity, among other results, are pointing out a cross-talk between effectors of the AA pathway (LOX and COX products) and PPAR*γ* activity. Deregulation of this cross-talk is thought to allow tumor progression.

## 5. OPTIMIZING THERAPEUTIC EFFECTS OF THE LOX/COX/PPAR*γ* CROSS-TALK

Despite the great effort in research in lung cancer basic biology and cancer early detection and prevention, there has been no significant improvement in five-year survival. As we have been previously described, several reports are showing that combination therapy against enzymes of the AA metabolism has a dramatic potential in chemoprevention and chemotherapy [21, 49, 51, 57]. Moreover, new drugs, such as Licofelone, designed to aim at a dual inhibition of the LOX and COX pathways, have proved to be effective in reducing growth in cancer cell lines. Interestingly, a recently published report, that uses a mathematical model to study the interactions of the AA metabolic network, has revealed that a dual inhibitor against LOX/COX is more effective than a combination of single COX and LOX inhibitors [84]. A successful attempt to reduce cell growth in cancer, through the AA metabolic pathway, may have great potency if involves inhibition of both the LOX and COX pathways and activation of PPAR*γ*. More research is needed in this subject to confirm the safety of dual inhibitors regarding GI toxicity and cardiovascular tolerability. In addition, efficiency of dual inhibitors in preventing cancer growth and inducing cell death has to be proved in other cancers especially in lung cancer.

To manage the systemic toxicity for lung cancer therapy, we have used a regional delivery strategy [85, 86]. With aerosolized drug delivery, there is direct delivery of the therapeutic agent to the target transforming cell population injured by chronic exposure tobacco smoke. Despite attractive results with relevant animal models and early clinical experience [87], this approach has not received serious commercial attention. However, aerosolized drug delivery has the appeal of avoiding certain important complications of PPAR*γ* activation, related to the complicated enterohepatic metabolism of these drugs [88].

Increasing evidence suggests that PPAR*γ* acts as a key control element of the AA pathway. Pawliczak et al. [66, 67] have shown that cPLA2*α*, responsible of the activation of AA, regulates PPAR*γ* expression and COX-2 and IL-8 expressions through PPAR*γ* in lung cancer cell lines. This last result is especially intriguing since it suggests that PPAR*γ* can also stimulate growth in addition of its role as suppressor of cell growth and inductor of apoptosis. Despite several evidence of the mainstream activity of PPAR*γ*, always in the context of an increased expression of the LOX pathway and/or COX-2, we should consider a more general role for PPAR*γ* as feedback regulator of the AA pathway.

In this context, using PPAR*γ* agonists, such as the TZDs, has provided a considerable amount of information about the PPAR*γ* function. But it has also revealed the great complexity of their indirect effects not related to PPAR*γ* [22, 23]. One approach to study PPAR*γ* function, without the interference of TZDs, would be to overexpress PPAR*γ* in NSCLC. Overexpression of PPAR*γ* has no effect on proliferation but it affects anchorage-independent growth, invasiveness and induces a differentiation from a mesenchymal to an epithelial-like phenotype [89]. In the same vein, some recent studies are suggesting a role for PPAR*γ* in control of anoikis by interacting with focal adhesion proteins [90, 91]. To better understand the PPAR*γ* function, a more direct approach might be to study the effect of overexpression and silencing of PPAR*γ* on mechanisms of cell proliferation and apoptosis. Generating such data could enable the development of a mechanistic model that could explain the inefficient response of PPAR*γ*, in the context of overexpression of the LOX/COX pathways, in lung cancer. This model may also allow a better understanding of LOX/COX interaction and PPAR*γ* function. As a result of this approach, a combination therapy, based on dual inhibitors of the LOX/COX pathways, could be developed in a more rationale fashion and provide a better result in chemoprevention and chemotherapy.

## 6. CONCLUSION

With considering the clinical significance of AA metabolism, it may be useful to partition certain aspects of this complex biology. For example, it is known that COX products are constitutively required for the maintenance of normal gastric epithelium, and from a clinical tolerance perspective, it would be good not to interfere with this function.

As we have discussed, chronic injury initiates an excessive release of cytokines and other inflammatory mediators such as arachidonic acid metabolism products which could trigger carcinogenesis. In devising therapeutic strategies, with the arachidonic acid products, greater attention to the impact of levels of mediators may be rewarding. For beneficial effects with carcinogenesis, it would be of interest to evaluate if reduction, rather than complete elimination, in levels of arachidonic acid products would be efficacious without incurring clinical toxicity. Moreover, exploring the effects of PPAR*γ* activity in different contexts, such as anoikis, could provide relevant mechanistic information about PPAR*γ* function that might allow an improved design of combination therapies.

Important opportunities exist to reduce the occurrence of low frequency but significant hepatic toxicity by considering the use of aerosolized drug delivery strategies. This would potentially greatly enable the use of combination approaches, as discussed in this review, for early lung cancer management applications.

Development of robust clinical pharmacology tools, such as the assay for urinary PGM, could allow for a precise and adaptive method to define optimal dosing for LOX/COX inhibitors. This approach may provide an important new opportunity in learning how to more effectively exploit the effects of inhibiting LOX and COX pathways, in combination with PPAR*γ* activation, on control of proliferation and apoptosis in lung cancer.
